# McKillip Veterinary College

**Published:** 1895-10

**Authors:** 


### McKILLIP VETERINARY COLLEGE.

The very pleasant trip by Eastern veterinarians to the McKillip Veterinary College at Chicago will ever be remembered as a bright episode of the Convention of 1895. Aside from the utmost effort on behalf of McKillip's officers to provide for our comfort and pleasure at a midway point in time of our journey to Des Moines, the longest to be remembered and the most grateful surprise to us all was to find so completely an equipped veterinary college, the munificence of an individual veterinarian. It made those feel good who fifteen or more years ago had sat upon the benches of other schools to note the great changes, the broad advances, the improved facilities, the stronger equipment, now than then, and to compare the arrangement of the facilities at McKillip with those in other institutions, and it was the consensus of opinion that, for a school confined within four walls, it certainly surpasses in arrangement any of the present schools. The lecture-rooms, laboratories, operating-room, reading-room, etc., for light, ventilation, cleanliness, and cheerfulness are all that one could desire, and must have a good influence on the scholars who will live as such for a time within her walls.

McKillip has a bright future before her. With the best of facilities, a liberal master at the head, and a field of great breadth, she should take the leading position of the central Western schools. She has done well in establishing a three-years' course at her birth; a broad curriculum; has already strengthened her faculty for the second-year's instruction, and with the same liberality in the future as has characterized this institution in the past, we are sure that she will soon draw around her the strongest faculty of any of the new schools, and will outstrip many of her older competitors. When this school has consummated the liberal plans on which it is based, drawn to her aid and support the strong faculty she deserves and can win, the founder, Dr. M. H. McKillip will have contributed a benefaction to the veterinary profession by his liberality that will live as a monument of honor to his name and fame long after his day and generation. As a successful every-day practitioner and friend among his clientage and people, he has won an honored place in his city and State, and is now destined to win equally as high a place among the profession of to-day and the future.

